# Molecular correlates of anemia in primary myelofibrosis: a significant and independent association with U2AF1 mutations

**DOI:** 10.1038/bcj.2016.22

**Published:** 2016-04-08

**Authors:** D Barraco, Y C Elala, T L Lasho, K H Begna, N Gangat, C Finke, C A Hanson, R P Ketterling, A Pardanani, A Tefferi

**Affiliations:** 1 Division of Hematology, Department of Medicine, Mayo Clinic, Rochester, MN, USA; 2 Division of Hematology, Azienda Ospedaliero-Universitaria Santa Maria della Misericordia, Udine, Italy; 3 Division of Hematopathology, Mayo Clinic, Rochester, MN, USA; 4 Division of Cytogenetics, Mayo Clinic, Rochester, MN, USA

Anemia is a cardinal manifestation of primary myelofibrosis (PMF) and constitutes a negative prognostic factor, being an independent risk factor for survival both in the international prognostic score system (IPSS)^1^ and dynamic IPSS (DIPSS)-plus.^2^ Anemia in myelofibrosis is also inversely correlated with patient-reported quality of life. Between 35 and 54% of patients with PMF are anemic at diagnosis with a hemoglobin level of <10 g/dl^1^ with the proportion increasing to 47–64% after about 1 year from the time of diagnosis.^3^

Pathogenesis of PMF-associated anemia is poorly understood and is multifactorial, involving hypersplenism, chronic low-grade hemolysis and ineffective hematopoiesis, the latter influenced by both abnormal expression of proinflammatory cytokines and other growth factors and, intrinsic erythroid cell defects.^3, 4, 5^

Current drugs, including JAK inhibitors, are suboptimal in the treatment of PMF-associated anemia and better information on its pathogenesis is critical for the development of more effective drugs. In the current study of *JAK2*/*CALR*/*MPL-*annotated patients with PMF, we examined the correlation between anemia with both driver and non-driver mutations,^6, 7^ as well as cytogenetic abnormalities, in order to gain better insight into its pathogenesis.

This study was approved by the institutional review board of Mayo Clinic (Rochester, MN, USA). Study patients were selected from our institutional database of myeloproliferative neoplasms based on the 2008 World Health Organization criteria diagnosis of PMF.^8^ Risk stratification was according to DIPSS-plus system.^2^ Screening for *JAK2*, *CALR*, *MPL*, *ASXL1*, *TET2*, *EZH2*, *IDH1*, *IDH2* and spliceosome (*SF3B1*, *U2AF1*, *SRSF2*, *ZRSR2*) mutations was performed according to previously described methods.^6, 7^ Statistical analyses considered clinical and laboratory data collected at the time of diagnosis or referral to the Mayo Clinic. Differences in the distribution of continuous variables between categories were analyzed by either Mann–Whitney or Kruskal–Wallis test. Patient groups with nominal variables were compared by *X*^2^ test. Survival analysis was considered from the date of diagnosis or referral to date of death (uncensored) or, last contact (censored). Survival curves were prepared by the Kaplan–Meier method and compared by the log-rank test. The Cox proportional hazard regression model was used for multivariate survival analysis. *P*<0.05 was considered significant. The Stat View (SAS Institute, Minneapolis, MN, USA) statistical package was used for all calculations.

A total of 722 PMF patients (median age was 64, and 64% were males) were studied. Presenting clinical and laboratory details are outlined in Table 1. Cytogenetic studies were available in 703 patients and included normal karyotype in 61% and normal or favorable abnormalities in 88%.^9^ Other mutations that were concomitantly analyzed included *ASXL1* (*n*=480), *SF3B1* (*n*=415), *U2AF1* (*n*=457), *SRSF2* (*n*=474), *TET2* (*n*=180), *EZH2* (*n*=374), *ZRSR2* (*n*=180), *IDH1* (*n*=187) and *IDH2* (*n*=187); their respective frequencies were 38, 8, 16, 15, 18, 4, 11, 5 and 7%.

Patients were stratified according to the grade of anemia as mild (for hemoglobin level ⩾10 g/dl but below sex-adjusted normal value), moderate (for hemoglobin level between 8 g/dl and <10 g/dl) and severe (hemoglobin <8 g/dl or transfusion-dependent). At time of referral, 628 (87%) patients had anemia, of which 295 (47%) were mild, 98 (16%) moderate and 235 (37%) severe.

In univariate analysis, anemia was significantly associated with advanced age (*P*<0.0001), lower leukocyte count (*P*<0.0001), lower platelet count (*P*<0.0001), presence of circulating blasts (*P*=0.0014), presence of constitutional symptoms (*P*<0.0001), driver mutational status (*P*<0.0001) and *U2AF1* mutation (*P*<0.0001). On multivariate analysis, advanced age (*P*=0.0002), lower platelet count (*P*=0.03), the presence of constitutional symptoms (*P*=0.04), absence of *JAK2* mutation (*P*=0.03), absence of *CALR* type 1/type 1-like mutation (*P*= 0.01) and presence of *U2AF1* (*P*<0.0001) retained significance.

At a median follow-up of 3 years (range 0–28.1 years), 439 (61%) deaths were recorded. In univariate analysis, the presence of *U2AF1* mutation is associated with inferior survival (*P*<0.0001; Figure 1). However, significance was lost when analysis was adjusted for age and anemia (*P*=0.24)

The current study confirms our previous observation regarding the association of mutant *U2AF1* with anemia,^10^ supporting its role in hematopoiesis^11^ and in the pathogenesis of PMF-associated anemia. Our observation also supports the need to screen patients with PMF for *U2AF1* mutations, in order to identify those who are at risk for the development of anemia, as well as those who might benefit from accordingly targeted therapy.^12^

## Figures and Tables

**Figure 1:**
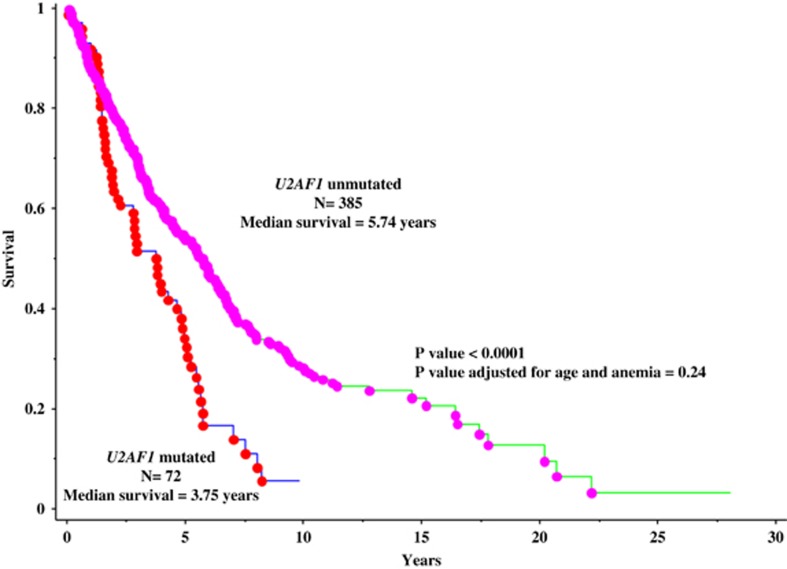
Overall survival of 457 patients with PML stratified by the presence or absence of U2AF1 mutations.

**Table 1:** Clinical and laboratory features of 722 patients with primary myelofibrosis patients stratified by grades of anemia

*Variables*	*All* (n*=722*)	*No anemia* (n*=94*)	*Mild anemia* (n*=295*)	*Moderate anemia* (n*=98*)	*Severe anemia* (n*=235*)	P-*value univariate*	P-*value multivariate*
Age at referral in years median (range)	64 (22–90)	60.5 (30–87)	61 (22–88)	64.5 (28–89)	69 (37–90)	**<0.0001**	**0.0002**
Male (%)	464 (64%)	55 (59%)	192 (65%)	61 (62%)	156 (66%)	0.55	
Leukocytes, × 10^9^/l median (range)	9 (0.8–236.1)	13.950 (2.8–4.1)	9.0 (1.1–176.0)	6.450 (1.7–236.1)	7.9 (0.8–218.5)	**<0.0001**	0.061
Platelets, × 10^9^/l median (range)	212.0 (10.0–466.0)	309.0 (15.0–288.0)	243.0 (13.0–255.0)	202.0 (14.0–282.0)	140.0 (10.0–2466.0)	**<0.0001**	**0.03**
Circulating blasts % median (range)	1 (0–15)	0 (0–5)	0 (0–15)	1 (0–8)	1 (0–15)	**0.0014**	0.36
Presence of constitutional symptoms *n* (%)	231 (32%)	20 (21%)	79 (27%)	31 (32%)	101 (43%)	**<0.0001**	**0.04**
Presence of palpable splenomegaly N evaluable=706 *n* (%)	514 (73%)	63 (68%)	208 (72%)	68 (72%)	175 (75%)	0.62	
DIPSS-plus^a^ risk N evaluable =703						**<0.0001**	
Low	94 (13%)	31 (34%)	61 (21%)	2 (2%)	0 (0%)		
Intermediate-1	119 (17%)	27 (29%)	85 (30%)	7 (7%)	0 (0%)		
Intermediate-2	259 (37%)	27 (29%)	109 (38%)	62 (64%)	61 (27%)		
High	231 (33%)	7 (8%)	29 (10%)	26 (27%)	169 (73%)		
Driver mutations						**<0.0001**	
*JAK2* mutated *n* (%)	476 (66%)	73 (78%)	175 (59%)	66 (67%)	162 (69%)		**0.03**
*CALR* type 1/type 1-like *n* (%)	115 (16%)	12 (13%)	70 (24%)	14 (14%)	19 (8%)		**0.01**
*CALR* type 2/type 2-like *n* (%)	24 (3%)	1 (1%)	14 (5%)	5 (5%)	4(2%)		
*MPL* mutated *n* (%)	38 (5%)	0 (0%)	18 (6%)	6 (6%)	14 (6%)		
Triple negative *n* (%)	69(10%)	8 (8%)	18 (6%)	7 (7%)	36 (15%)		
							
*Cytogenetic categories N evaluable =703 (97%)*							
Normal	426 (61%)	64 (70%)	173 (61%)	56 (58%)	133 (58%)		
Normal vs abnormal						0.24	
Favorable	620 (88%)	82 (89%)	255 (90%)	87 (90%)	196 (85%)		
Favorable vs unfavorable						0.40	
*ASXL1* N evaluable=480	181(38%)	21 (31%)	70 (36%)	28 (42%)	62 (41%)	0.50	
*SF3B1* N evaluable=415	35 (8%)	4 (7%)	11 (7%)	6 (10%)	14 (10%)	0.64	
*U2AF1* N evaluable=457	72 (16%)	2 (3%)	15 (8%)	11 (18%)	44 (30%)	**<0.0001**	**<0.0001**
*SRSF2* N evaluable=474	70 (15%)	9 (13%)	24 (13%)	6 (9%)	31 (20%)	0.09	
*TET2* N evaluable=180	32 (18%)	2 (7%)	13 (19%)	7 (30%)	10 (16%)	0.21	
*EZH2* N evaluable=374	16 (4%)	2 (4%)	8 (5%)	2 (4%)	4 (3%)	0.85	
*ZRSR2* N evaluable=180	19 (11%)	1 (4%)	11 (16%)	0 (0%)	7 (11%)	0.11	
*IDH1* N evaluable=187	9 (5%)	1 (4%)	3 (4%)	2 (8%)	3 (5%)	0.87	
*IDH2* N evaluable=187	14 (7%)	1 (4%)	5 (7%)	1 (4%)	7 (11%)	0.58	

Abbreviation: DIPSS-plus, dynamic international prognostic scoring system-plus.

a DIPSS-plus:5 DIPSS-plus uses eight independent predictors of inferior survival: age >65 years, hemoglobin <10 g/dl, leukocytes >25 × 109/l, circulating blasts ⩾1%, constitutional symptoms, red cell transfusion dependency, platelet count <100x109/l and unfavorable karyotype (that is, complex karyotype or sole or two abnormalities that include þ8, −7/7q−, i(17q), inv,3 −5/5q−, 12p− or 11q23 rearrangement). The presence of 0, 1, ‘2 or 3' and 4 adverse factors defines low, intermediate-1, intermediate-2 and high-risk disease. Statistically significant *P*-values are in bold.
